# Calcium requirements in growing Japanese quail from 21 to 35 days post-hatch

**DOI:** 10.1016/j.psj.2024.104700

**Published:** 2024-12-20

**Authors:** Fahimeh Pourmollaei, Mahmoud Ghazaghi, Mohammad Rokouei, Farzad Bagherzadeh-Kasmani, Mehran Mehri

**Affiliations:** Department of Animal Sciences, Faculty of Agriculture, University of Zabol, Sistan, 98661-5538, Iran

**Keywords:** Bone health, Breaking strength, Performance, Seedor index, Spline model

## Abstract

An experiment was conducted to estimate the optimal calcium (Ca) requirement for growth performance and bone health in quail from 21 to 35 days posthatch. Five dietary treatments containing 0.45, 0.60, 0.75, 0.90, and 1.05 % Ca were evaluated in a completely randomized design with 6 replicates per treatment and 12 birds per pen. Body weight gain (BW gain; *P* = 0.04), tibia-Ca (*P* = 0.01), tibia ash (*P* = 0.01), and bone breaking strength (BBS; *P* = 0.01) increased quadratically with increasing dietary Ca levels, while feed conversion ratio (FCR) showed a tendency to decrease (*P* = 0.07). Based on the statistical model, the optimal Ca requirements for BW gain and FCR were estimated to range from 0.75 to 0.84 % and 0.74 to 0.83 %, respectively. The Ca requirements for the Seedor index, tibia mass, and tibia length were estimated to range from 0.83 to 0.87 %, 0.81 to 0.87 %, and 0.78 to 0.86 %, respectively. The corresponding values for tibia-Ca, tibia ash, and BBS ranged from 0.67 to 0.73 %, 0.68 to 0.73 %, and 0.75 to 0.83 %, respectively. The study revealed that Ca requirements for optimal bone density may exceed those for growth performance, indicating the need for dietary Ca recommendations to be influenced by bone health considerations. These findings suggest that dietary calcium levels between 0.75 % and 0.87 % are optimal for growth performance and bone health in quail during the post-hatch growth period, with higher levels potentially required to maximize bone density and strength.

## Introduction

Calcium (Ca) is a vital mineral in poultry nutrition, playing a crucial role in various physiological processes, including bone development, muscle contraction, and egg production. In growing Japanese quail (*Coturnix japonica*), adequate Ca intake is important as it not only supports optimal growth performance but also contributes to the structural integrity of bones (Perine et al., 2016; Reddy, et al., 1980). Furthermore, studies have shown that Ca plays a critical role in metabolic functions such as blood coagulation and neurotransmission, which are essential for maintaining the health of growing birds (Stanquevis, et al., 2021).

As quail are increasingly recognized for their economic value in both meat and egg production, understanding their specific dietary needs has become essential for maximizing their health and productivity. Despite the significance of Ca in avian diets, there remains a gap in the literature regarding the precise Ca requirements for growing quail. Previous studies have primarily focused on other poultry species, leading to a reliance on generalized nutritional guidelines that may not apply to quail (Perine, et al., 2022). Research indicates that inadequate Ca levels can lead to poor bone mineralization and increased susceptibility to fractures, which can adversely affect overall growth and production efficiency (Ribeiro, et al., 2016). Given these considerations, it is imperative to conduct targeted research to establish specific Ca requirements for Japanese quail during their growth phase.

Concerns are increasing regarding the accuracy of the reported Ca requirements for quails, as some studies use inappropriate statistical models. For example, Reddy, et al. (1980) employed an analysis of variance followed by multiple range test comparisons to estimate Ca needs of quail chicks. This methodology can result in misleading conclusions, potentially leading to either an underestimation or overestimation of the actual Ca requirements (Pesti, et al., 2009). This situation highlights the need for more robust modeling techniques to accurately determine nutrient needs in growing quail chicks.

This study aims to estimate the Ca requirements of growing Japanese quail by using growth performance and bone health indicators as response variables, with the goal of optimizing feed formulation and enhancing both productivity and welfare in commercial settings.

## Materials and methods

### Ethics statement

This study protocol received approval from the Research Animal Ethics Committee at the University of Zabol and complies with the regulations of the Iranian Council of Animal Care. All experimental procedures followed the guidelines established by ARRIVE and the National Institutes of Health for animal research (Du Sert, et al., 2020).

### Chemical ANALYSIS

Feed ingredient samples were analyzed for dry matter (DM; method 930.15, AOAC, 2006), ash content (method 942.05, AOAC, 2006), crude fiber (method 978.10, AOAC, 2006), ether extract (method 2003.05, AOAC, 2006), Ca (method 934.01, AOAC (2006), phosphorus (method 965.17, AOAC, 2006), and crude protein (CP; method 990.03, AOAC, 2006).

### Birds and experimental diets

A dose-response experiment was conducted on growing Japanese quail from days 21 to 35 to estimate Ca requirements. Day-old chicks were obtained from the experimental farm at the University of Zabol, Iran. From hatching until 20 days of age, they were fed a standard diet formulated to meet the nutrient requirements of growing Japanese quails, based on NRC (1994) recommendations. A basal diet was formulated to meet all nutritional requirements of growing quails except for Ca (Table 1), and five experimental diets containing 0.45 % to 1.10 % Ca, with increments of 0.15 %, were developed. On day 21, a total of 360 birds were weighed and randomly distributed into 5 experimental treatments, with each treatment comprising 6 replicate pens, containing 12 birds per pen. Feed and water were provided ad libitum throughout the experiment. The room temperature and humidity were maintained at 24 ± 1.20 °C and 60 ± 3.5 %, respectively. Lighting program was 18L:6D throughout the experiment.

**Table 1:** Composition of the experimental diets.

Ingredient	Dietary calcium (%)				
	0.45	0.60	0.75	0.90	1.05
Corn, grain	53.21	53.21	53.21	53.21	53.21
Soybean Meal (44 %)	35.00	35.00	35.00	35.00	35.00
Corn gluten meal (60 %)	5.31	5.31	5.31	5.31	5.31
Sand	2.42	2.03	1.63	1.24	0.84
Soybean oil	1.00	1.00	1.00	1.00	1.00
Di-calcium phosphate	0.89	0.89	0.89	0.89	0.89
Limestone	0.39	0.78	1.18	1.57	1.97
NaHCO_3_	0.33	0.33	0.33	0.33	0.33
DL-Methionine	0.33	0.33	0.33	0.33	0.33
NaCl	0.29	0.29	0.29	0.29	0.29
Mineral Premix^1^	0.25	0.25	0.25	0.25	0.25
Vitamin Premix^2^	0.25	0.25	0.25	0.25	0.25
L-Lysine HCl	0.23	0.23	0.23	0.23	0.23
L-Threonine	0.10	0.10	0.10	0.10	0.10
Nutrient composition					
AME (Kcal/kg)	2920	2920	2920	2920	2920
CP (%)	25.0	25.0	25.0	25.0	25.0
Total Lysine (%)	1.38	1.38	1.38	1.38	1.38
Total Methionine + Cysteine (%)	1.11	1.11	1.11	1.11	1.11
Total Methionine (%)	0.72	0.72	0.72	0.72	0.72
Total Tryptophan (%)	0.27	0.27	0.27	0.27	0.27
Total Arginine (%)	1.54	1.54	1.54	1.54	1.54
Total Threonine (%)	1.02	1.02	1.02	1.02	1.02
Ca (%)	0.45	0.60	0.75	0.90	1.05
P available (%)	0.35	0.35	0.35	0.35	0.35
Na (%)	0.23	0.23	0.23	0.23	0.23
Cl (%)	0.26	0.26	0.26	0.26	0.26
K (%)	0.88	0.88	0.88	0.88	0.88
DEB^3^ (mEq/kg)	250	250	250	250	250

1 Mineral premix provided per kilogram of diet: Mn (from MnSO4·H2O), 65 mg; Zn (from ZnO), 55 mg; Fe (from FeSO4·7H2O), 50 mg; Cu (from CuSO4·5H2O), 8 mg; I [from Ca (IO3)2·H2O], 1.8 mg; Se, 0.30 mg; Co (from Co2O3), 0.20 mg; Mo, 0.16 mg.

2 Vitamin premix provided per kilogram of diet: vitamin A (from vitamin A acetate), 11,500 IU; cholecalciferol, 2,100 IU; vitamin E (from dl-α-tocopheryl acetate), 22 IU; vitamin B12, 0.60 mg; riboflavin, 4.4 mg; nicotinamide, 40 mg; calcium pantothenate, 35 mg; menadione (from menadione dimethylpyrimidinol), 1.50 mg; folic acid, 0.80 mg; thiamine, 3 mg; pyridoxine, 10 mg; biotin, 1 mg; choline chloride, 560 mg; ethoxyquin, 125 mg.

3 Dietary Electrolyte Balance: represents dietary Na +*K* − Cl in mEq/kg of diet.

### Performance

Body weight, body weight gain (BW gain), and feed intake (FI) were measured on a cage basis from day 21 to day 35, and the feed conversion ratio (FCR) was calculated accordingly. No mortality was observed during the study.

### Serum Ca

On d 35, blood samples were collected from the wing vein of quails using sterile syringes. After allowing the blood to clot for 30 min at room temperature and centrifuging at 3,000 rpm for 10 min, serum Ca was measured using a commercial kit from Pars-Azmoun (Tehran, Iran).

### Bone characteristics

At the end of the experiment, two birds per replicate were euthanized, and tibiae were collected. Both tibiae from each bird were removed, cleaned of soft tissues (muscle, fat, tendons, etc.), and stored at –20°C in individual plastic bags until further analysis. Cleaned tibiae were boiled and thoroughly cleaned of any remaining tissue, then weighed, dried at 109°C for 12 h, chemically cleaned in acetone for 48 h, dried again at 109°C for another 12 h, and finally ashed overnight in a muffle furnace at 550°C to determine ash content, following the procedure outlined by Asensio, et al. (2020). Ca and phosphorus content were analyzed using AOAC (2006) methods. Bone (tibia) breaking strength (BBS) was measured using a 3-point bending test with a material testing machine (TA.XTplus100, Stable Micro Systems, Godalming, United Kingdom), following the method described by Alfonso-Carrillo, et al. (2021). Bone length and weight were measured using a digital caliper and a digital scale. These measurements were used to calculate the Seedor index (SI), which is an indicator of bone mineral density, by dividing bone weight by its length (Seedor, et al., 1991).

### Statistical analysis

The data were analyzed using the GLM procedure in SAS (2002) to evaluate the linear and quadratic responses of the birds to dietary Ca, with the average value of each pen serving as the experimental unit. To determine Ca requirements, various broken-line regression models were applied according to Khosravi, et al. (2016) as follows:

One-slope linear ascending (or descending) broken line:

Y=L+U×(R−X)×(X<R)

Two-slope broken line:

Linear ascending (or descending)-linear descending (or ascending):

Y=L+U×(R−−X)×(X<R)+V×(X−−R)×(X>R)

Quadratic ascending (or descending)-linear descending (or ascending):

$$Y=L+U\times {\left(R--X\right)}^{2}\times \left(X<R\right)+V\times \left(X--R\right)\times \left(X>R\right)$$

Quadratic ascending (or descending)-quadratic descending (or ascending):

$$Y=L+U\times {\left(R--X\right)}^{2}\times \left(X<R\right)+V\times {\left(X--R\right)}^{2}\times \left(X>R\right)$$

where Y represents the bird response; L is the asymptote for the first segment; U and V are the slopes for the first and second lines, respectively, indicating either an increasing or decreasing trend; and R is the breakpoint regarded as the "requirement" point.

The most suitable model for each variable response was chosen based on the highest coefficient of determination (R²) and the lowest standard deviation of the residuals (S*_y._ₓ*), which is also known as the standard deviation of the data points around a fitted line.

$$S_{y.x}=\;\sqrt{\frac{SS}{df}}$$

A two-tailed one-sample t-test was employed to assess whether the estimated Ca requirements for each response significantly differed from the NRC (1994) recommendations for the Ca requirements of quail chicks (GraphPad 10.2.3, Boston, Massachusetts USA).

## Results

### Analysis of variance

No mortality and bone abnormality were observed during the study. Performance data was shown in Table 2. No significant effect was observed on FI, while BW gain quadratically increased with increasing dietary Ca (*P* = 0.048). Feed conversion ratio tended to be decreased with increasing dietary Ca (*P* = 0.076). Increasing dietary Ca did not affect serum-Ca (Table 3), tibia mass, tibia length, and SI (Table 4), while quadratically increased tibia-Ca (*P* = 0.013), tibia ash (*P* = 0.014), and BBS (*P* = 0.006; Table 4). As shown in Table 5, strong positive correlation was observed between tibia ash and BBS (*r* = 0.95; *P* < 0.001), tibia-Ca and BBS (*r* = 0.94; *P* < 0.001), and tibia mass and SI (*r* = 0.95; *P* < 0.001).

**Table 2:** Growth performance in growing quail chicks fed gradient levels of dietary Ca.

Response	Dietary Ca (%)					SEM	Probability	
	0.45	0.60	0.75	0.90	1.05		Linear	Quadratic
Feed intake (g/b/d)	30.9	31.2	31.9	31.8	31.0	0.53	0.614	0.157
Gain (g/b/d)	7.91	8.17	8.11	8.02	7.09	0.31	0.080	0.048
Feed conversion ratio	3.93	3.92	3.76	4.00	4.40	0.51	0.076	0.201

**Table 3:** Concentration of serum Ca in growing quail chicks fed gradient levels of dietary Ca.

Response	Dietary Ca (%)					SEM	Probability	
	0.45	0.60	0.75	0.90	1.05		Linear	Quadratic
Serum Ca (mg/dL)	10.76	10.91	10.95	11.06	11.07	0.98	0.804	0.953

**Table 4:** Analysis of variance of bone attributes in growing quail chicks fed gradient levels of dietary Ca.

Response	Dietary Ca (%)					SEM	Probability	
	0.45	0.60	0.75	0.90	1.05		Linear	Quadratic
Tibia mass (g)	0.977	0.983	0.985	0.981	0.955	0.02	0.466	0.349
Tibia length (mm)	55.16	55.23	55.19	55.11	54.65	0.32	0.274	0.369
Seedor index (mg/mm)	17.70	17.78	17.85	17.81	17.47	0.34	0.691	0.471
Tibia ash (%)	36.25	37.27	37.53	37.05	36.67	0.37	0.601	0.014
Tibia Ca (%)	19.01	19.69	19.87	19.54	19.29	0.25	0.611	0.013
Bone breaking strength (KgF)	2.39	2.54	2.58	2.56	2.46	0.05	0.406	0.006

**Table 5:** Correlation matrix of bone attributes in growing quail chicks fed gradient levels of dietary Ca.

	Tibia mass	Tibia length	Seedor index	Tibia ash	Tibia Ca	BBS	Serum Ca
Tibia mass	1.00	0.25	0.95	0.48	0.48	0.47	0.29
		***P* = 0.05**	***P* <.0001**	***P* = 0.001**	***P* <.0001**	***P* = 0.001**	***P* = 0.03**
Tibia length	0.25	1.00	−0.04	−0.04	−0.01	0.01	0.17
	***P* = 0.05**		*P* = 0.74	*P* = 0.75	*P* = 0.93	*P* = 0.93	*P* = 0.21
Seedor index	0.95	−0.04	1.00	0.51	0.50	0.49	0.24
	***P* <.0001**	*P* = 0.74		***P* <.0001**	***P* <.0001**	***P* <.0001**	*P* = 0.06
Tibia ash	0.48	−0.04	0.51	1.00	0.99	0.95	0.29
	***P* = 0.001**	*P* = 0.75	***P* <.0001**		***P* <.0001**	***P* <.0001**	***P* = 0.03**
Tibia Ca	0.48	−0.01	0.50	0.99	1.00	0.94	0.30
	***P* <.0001**	*P* = 0.93	***P* <.0001**	***P* <.0001**		***P* <.0001**	***P* = 0.02**
BBS	0.47	0.01	0.49	0.95	0.94	1.00	0.28
	***P* = 0.001**	*P* = 0.93	***P* <.0001**	***P* <.0001**	***P* <.0001**		***P* = 0.03**
Serum Ca	0.29	0.17	0.24	0.29	0.30	0.28	1.00
	***P* = 0.03**	*P* = 0.21	*P* = 0.06	***P* = 0.03**	***P* = 0.02**	***P* = 0.03**	

### Estimation of requirements

The optimal Ca requirements for various traits were estimated using one and two-slope broken line models. The best estimation was identified based on the minimum *S_y.x_* and maximum R^2^.

**Fig. 1:**
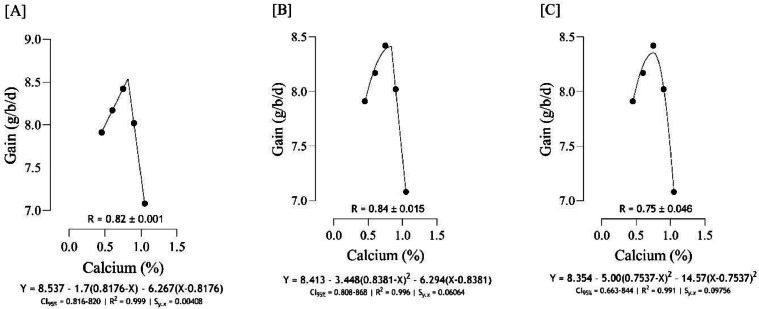
Estimation of the Ca requirements (R ± SE) for body weight gain (g/b/d) using two-slope linear-ascending linear-descending [A], two-slope linear-ascending quadratic-descending [B], and two-slope quadratic-ascending quadratic-descending [C] broken line models.

**Fig. 2:**
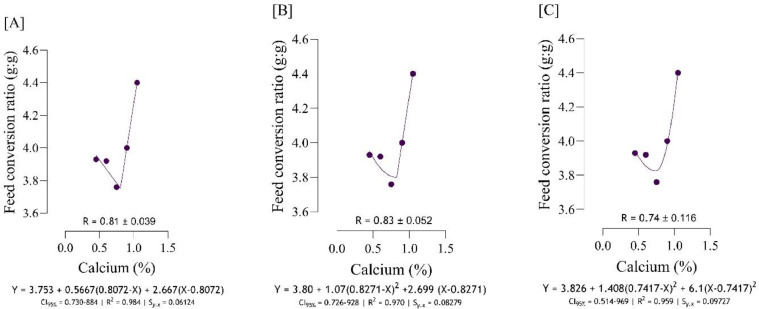
Estimation of the Ca requirements (R ± SE) for feed conversion ratio (g/g) using two-slope linear-descending linear-ascending [A], two-slope linear-descending quadratic-ascending [B], and two-slope quadratic-descending quadratic-ascending [C] broken line models.

**Fig. 3:**
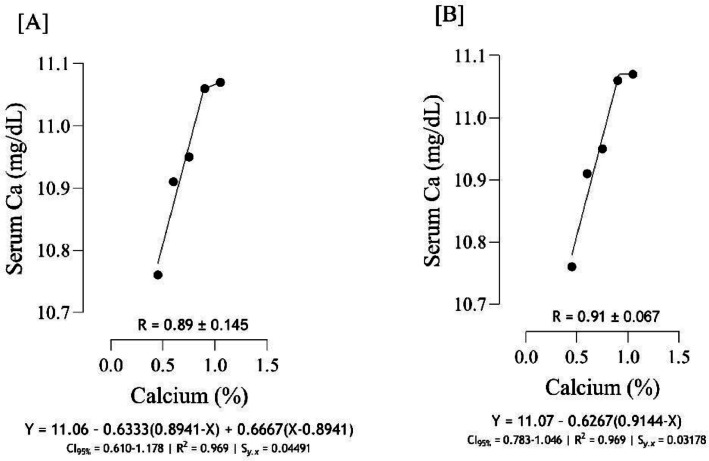
Estimation of the Ca requirements (R ± SE) for serum- Ca using two-slope linear-ascending linear-descending [A], and one-slope linear-ascending [B] broken line models.

The Ca requirement for BW gain (Fig. 1) was estimated to range from 0.75 to 0.84 %, and the best estimation was obtained at 0.82 % (*S_y.__x_* = 0.004082 | R^2^ = 0.999). The Ca requirement for FCR (Fig. 2), was estimated to range from 0.74 to 0.83 %, and the best estimation was obtained at 0.821 % (*S_y.__x_* = 0.06124 | R^2^ = 0.984). Serum-Ca (Fig. 3) was maximized at 0.91 % of dietary Ca (*S_y.__x_* = 0.03178 | R^2^ = 0.969).

**Fig. 4:**
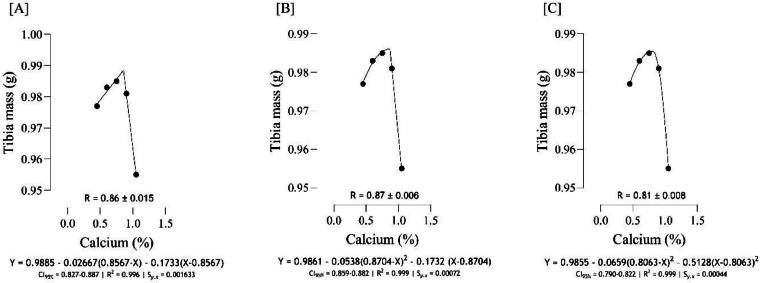
Estimation of the Ca requirements (R ± SE) for tibia mass (g) using two-slope linear-ascending linear-descending [A], two-slope linear-ascending quadratic-descending [B], and two-slope quadratic-ascending quadratic-descending [C] broken line models.

**Fig. 5:**
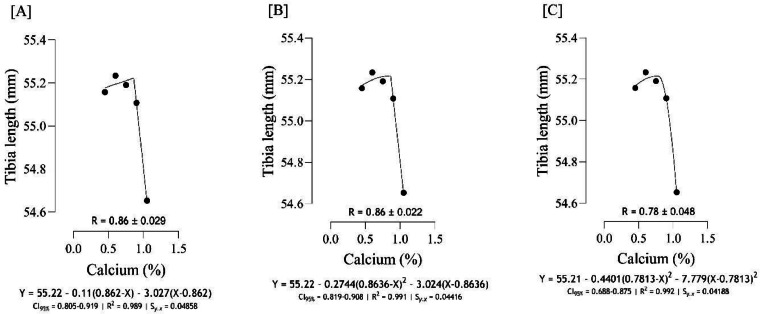
Estimation of the Ca requirements (R ± SE) for tibia length (mm) using two-slope linear-ascending linear-descending [A], two-slope linear-ascending quadratic-descending [B], and two-slope quadratic-ascending quadratic-descending [C] broken line models.

**Fig. 6:**
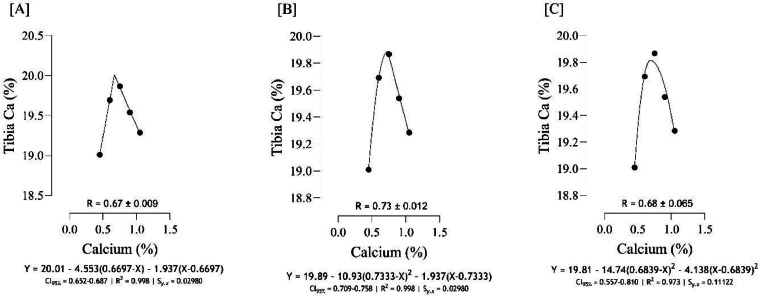
Estimation of the Ca requirements (R ± SE) for tibia-Ca (%) using two-slope linear-ascending linear-descending [A], two-slope linear-ascending quadratic-descending [B], and two-slope quadratic-ascending quadratic-descending [C] broken line models.

**Fig. 7:**
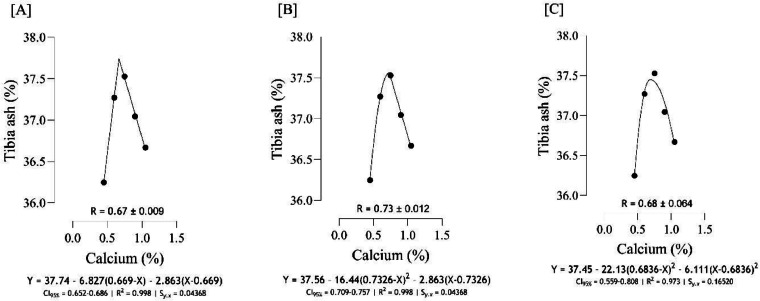
Estimation of the Ca requirements (R ± SE) for tibia ash (%) using two-slope linear-ascending linear-descending [A], two-slope linear-ascending quadratic-descending [B], and two-slope quadratic-ascending quadratic-descending [C] broken line models.

**Fig. 8:**
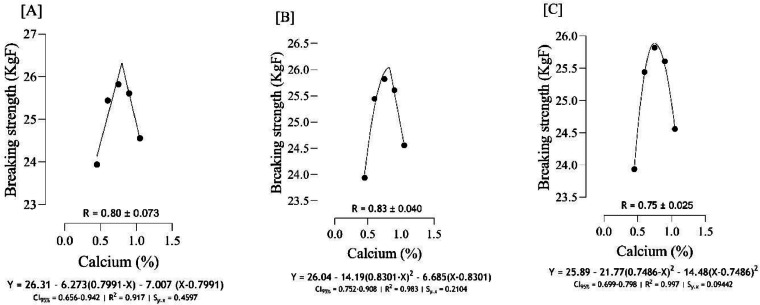
Estimation of the Ca requirements (R ± SE) for bone breaking strength (BBS; KgF) using two-slope linear-ascending linear-descending [A], two-slope linear-ascending quadratic-descending [B], and two-slope quadratic-ascending quadratic-descending [C] broken line models.

**Fig. 9:**
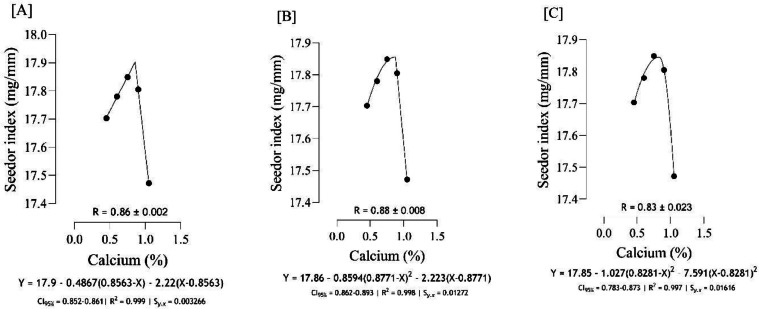
Estimation of the Ca requirements (R ± SE) for Seedor index (mg/mm) using two-slope linear-ascending linear-descending [A], two-slope linear-ascending quadratic-descending [B], and two-slope quadratic-ascending quadratic-descending [C] broken line models.

The optimal Ca for maximizing tibia mass (Fig. 4) was estimated to range from 0.81 to 0.87 %, and the best estimation was obtained at 0.87 % (*S_y.__x_* = 0.0004 | R^2^ = 0.999). Based on different broken line regressions, tibia length was maximized at 0.78 and 0.86 % Ca (Fig. 5), and the best estimate was obtained at 0.78 % Ca (*S_y.__x_* = 0.04188 | R^2^ = 0.992). Using three broken line models, the estimated values for maximizing both tibia-Ca (Fig. 6) and tibia ash (Fig. 7) were 0.67, 0.68, and 0.73 % and the best estimation for these traits was 0.73 % (*S_y.__x_* = 0.02980 | R^2^ = 0.998 and *S_y.__x_* = 0.04368 | R^2^ = 0.998, respectively). The BBS (Fig. 8) was maximized at 0.75, 0.80, 0.83 % of dietary Ca, and the best estimation was 0.75 % (*S_y.__x_* = 0.00442 | R^2^ = 0.997). The Ca requirements for maximizing SI (Fig. 9) were estimated at 0.83, 0.86, and 0.88 %, and the best estimation was 0.86 % (*S_y.__x_* = 0.003266 | R^2^ = 0.999).

## Discussion

Ca is an essential mineral crucial for various physiological functions, particularly bone development and growth performance in meat-type quail aged 15 to 35 days. During the early stages of life, quail chicks accumulate a substantial portion of their total calcium reserves, emphasizing its importance in this period (Perine, et al., 2016). Bagheri, et al. (2022) notes that Ca deficiency can lead to irreversible consequences on bone health and overall growth performance, reinforcing the need for adequate dietary Ca. Our findings highlight a distinct separation between Ca needs for skeletal integrity and those supporting growth performance in quail chicks. In addition, we demonstrated that modern strains of Japanese quail may need more dietary Ca than that recommended by NRC (1994) for growing period (0-6 week). This distinction is crucial for poultry nutritionists and producers aiming to optimize the health and productivity of their flocks, as the genetic progress in the modern strains of growing birds makes it necessary to update the nutritional requirements of the modern flocks. For example, Miller (1967) reported that growing Japanese quails with 0.44 % Ca showed a satisfactory BW gain, while in the present study, we showed that the optimal Ca level for maximum BW gain could be 0.82 % of diet, which was higher than recommended by NRC (1994). Understanding these differences can lead to better dietary formulations that support both skeletal integrity and overall growth.

We showed in the present study that the optimal Ca for growth performance and bone characteristics was not identical. While sufficient Ca is necessary for bone health, its relationship with growth performance is more complex, and there was no significant correlation between performance and bone density. Studies have shown that higher Ca levels do not always correlate with improved growth rates. For instance, while a diet containing approximately 0.90 % Ca may optimize bone health, bone strength, and mineralization during critical growth phases, lower levels may suffice for BW gain ranging from 0.73 to 0.78 % (Albino and de Toledo Barreto, 2003). The same result was observed by Edache, et al. (2005), showing that dietary levels of 0.50 and 1.0 % Ca will be adequate for maximum BW gain and bone ash of quail chicks, respectively, in the first 6 weeks of life. This distinction highlights the need for producers to prioritize skeletal health over immediate growth metrics when formulating diets for young quails. Ensuring adequate Ca intake during early life stages not only supports bone integrity but also lays the groundwork for future productivity as these birds mature.

One of the most important issues concerning nutritional requirements is the statistical approach to estimate the “requirement.” A comprehensive review by Pesti, et al. (2009) describes different methods to estimate nutritional requirements from experimental data, stressing the use of appropriate regression equations to “estimate” rather than “determine” the nutritional needs of nutrients in animal bioassays. For example, Reddy, et al. (1980) reported that Ca requirements for growing Japanese quails may not exceed 0.5 % of diet using multiple range tests. However, re-analysis of those data with regression equations revealed that the optimal Ca for growing quails fed 0.5 to 0.7 % P ranged from 0.8 to 0.9 % of diet, highlighting the importance of using the appropriate method to analyze data. An important, often overlooked, aspect of estimating nutritional requirements is using critical statistics, such as standard error of the estimate (SE of break point) and *S_y.x_* (standard deviation of the residuals), which can be used for calculating confidence interval (CI) and model precision, respectively. Standard error and CI calculations at the Ca requirement breakpoint add precision to our estimates, allowing for a nuanced interpretation that adapts to real-world variability. Since the estimated value as the “*requirement*” is not a static figure and could change under different conditions, it should be reported with associated SE and CI. As indicated in the present study, we used different broken line models and identified the best estimation of Ca requirements using the *S_y.x_* of the model. The next important terminology in the context of nutrient requirements is the misuse of “*determination*” instead of “*estimation*.” The former term describes a fixed value, while the latter indicates a flexible value with statistical merits. Using multiple range tests in dose-response studies results in “*determination*” of the required level of a nutrient without SE and CI, whereas regression functions provide an “*estimation*” of the optimal level of a nutrient along with SE and CI.

As indicated in the present study, we used the SE and CI of the breakpoint, as the requirement of Ca, to compare the estimated values with NRC (1994)recommendations. When the NRC (1994) recommended Ca level for growing quail chicks (0.80 %) falls outside the calculated CI (Fig. 1 to 9) based on the SE of requirement estimates, it indicates that the estimated values for Ca are statistically different from the NRC (1994) recommendation. The present research indicates that Ca requirements are higher for bone development (i.e., tibia mass and tibia length) and bone density (i.e., Seedor index) than that required for BW gain, which are higher than the levels recommended by NRC (1994) for growing quail chicks. However, the estimated Ca needs for maximum bone mineralization (i.e., tibia ash and tibia-Ca) were lower than the Ca recommendation by NRC (1994). Growth performance, especially in young quail, involves a broader range of physiological processes that depend on adequate Ca. These include not only skeletal development but also muscle function, enzyme activity, and cellular metabolism, all of which support optimal growth (Bagheri, et al., 2022; Stanquevis, et al., 2021). In contrast, tibia ash and tibia-Ca primarily reflect Ca deposited in the bone, which tends to stabilize at lower Ca levels once the critical threshold for bone mineralization is met. Therefore, a slight reduction in Ca may still allow for sufficient bone mineralization while not supporting the maximum growth performance, and bone mineralization may reach a plateau at lower Ca levels. In other words, higher Ca intake not only supports skeletal growth but also sustains essential physiological processes like muscle contraction, enzyme activity, and metabolic functions, which collectively promote optimal growth performance in quail (Kheiri and Landy, 2020).

Choi, et al. (2013) suggested that the body tightly regulates blood Ca levels, prioritizing bone mineralization to maintain structural integrity. This may occur at the expense of other growth processes when Ca intake is suboptimal. However, it should be noted that although bone mineralization was maximized at lower levels of dietary Ca, bone development and density were maximized at higher levels of dietary Ca. Souza, et al. (2017) reported that higher Ca levels are beneficial for SI in pullets, providing evidence that Ca requirements for bone development and density may be higher than those needed for bone mineralization.

In conclusion, recognizing the distinct Ca requirements for bone health versus growth performance is essential for effective quail dietary management. While both aspects are important, prioritizing skeletal health through adequate dietary Ca intake during critical developmental phases will ultimately enhance overall productivity and well-being within flocks. This study revealed that updating the nutritional requirements for modern quail strains may be warranted.

## Declaration of competing interest

The authors whose names are listed immediately below certify that they have NO affiliations with or involvement in any organization or entity with any financial interest (such as honoraria; educational grants; participation in speakers’ bureaus; membership, employment, consultancies, stock ownership, or other equity interest; and expert testimony or patent-licensing arrangements), or non-financial interest (such as personal or professional relationships, affiliations, knowledge or beliefs) in the subject matter or materials discussed in this manuscript.
